# Pain, Sleep, and Health-Related Quality of Life after Multidisciplinary Intervention for Chronic Pain

**DOI:** 10.3390/ijerph181910233

**Published:** 2021-09-28

**Authors:** Hafdís Skúladóttir, Herdis Sveinsdottir, Janean E. Holden, Thóra Jenný Gunnarsdóttir, Sigridur Halldorsdottir, Amalia Björnsdottir

**Affiliations:** 1 School of Health Sciences, University of Akureyri, Solborg v/Nordurslod, 600 Akureyri, Iceland; sigridur@unak.is; 2 School of Health Sciences, Faculty of Nursing, University of Iceland, Eiríksgata 34, 101 Reykjavík, Iceland; herdis@hi.is (H.S.); thoraj@hi.is (T.J.G.); 3 School of Nursing, University of Michigan, Ann Arbor, MI 48109, USA; holdenje@umich.edu; 4 School of Education, Faculty of Education and Pedagogy, University of Iceland, Stakkahlíð 1, 105 Reykjavík, Iceland; amaliabj@hi.is

**Keywords:** chronic pain, rehabilitation, health-related quality of life, sleep

## Abstract

Multidisciplinary pain-management programs have the potential to decrease pain intensity, improve health-related quality of life (HRQOL), and increase sleep quality. In this longitudinal prospective cohort study, the aim was to investigate the long-term effects of multidisciplinary pain rehabilitation interventions in Iceland. More precisely, we (a) explored and described how individuals with chronic pain evaluated their pain severity, sleep, and HRQOL at pre-treatment and at one-year follow-up and (b) examined what predicted the participants’ one-year follow-up HRQOL. Seventy-nine patients aged 20–68 years, most of whom were women (85%), responded. The participants scored their pain lower at one-year follow-up (*p* < 0.001). According to their response, most of them had disrupted sleep, mainly because of pain. One year after the treatment, more participants slept through the night (*p* = 0.004), and their HRQOL increased. Higher pre-treatment mental component summary (MCS) scores and having pursued higher education predicted higher MCS scores at one-year follow-up, and higher pre-treatment physical component summary (PCS) scores predicted higher PCS scores at one-year follow-up. Sleep problems, being a woman, and having children younger than 18 years of age predicted lower MCS scores at one-year follow-up. These findings are suggestive that patients should be examined with respect to their mental status, and it could be beneficial if they received some professional support after completing the intervention.

## 1. Introduction

Chronic pain has been defined as pain that persists beyond normal tissue healing time [1] and typically lasts longer than three months. Pain is the second largest contributor to disability worldwide, with low back pain being the single leading cause of disability [2]. The incidence, prevalence, severity, and accompanying impairments of chronic pain are among the main reasons for regarding pain as a public health priority, and for millions of people, pain is an inescapable reality of life [1,3]. For example, in Iceland, the prevalence of chronic pain is as high as 48%, and of those with chronic pain, approximately 30% experienced constant pain. Such pain levels affect every aspect of functioning [4].

Multidisciplinary long-term pain rehabilitation, also called interdisciplinary pain rehabilitation, uses a team of health-care professionals and an integrated approach to treat patients with non-malignant pain. Such programs are a combination of psychological interventions and physical training for situations where pharmacological treatment or physiotherapy are insufficient [5].

Health-related quality of life (HRQOL) describes the impact of health on people’s ability to function and participate in meaningful activities within the family, workplace, and community [6,7]. Measuring HRQOL is an important outcome in studies of patients with chronic pain [6,8,9] and is another way to assess patients’ subjective perspectives on their pain experience and its impact on their lives [6].

The finding from multidisciplinary long-term pain rehabilitation studies using HRQOL assessment are mixed. For example, patients with chronic pain benefit from multidisciplinary pain management programs in terms of better functioning, but the impact on pain was lower than expected [5]. Salathé et al.’s systematic review showed that long-term pain rehabilitation produces either no long-term increase in HRQOL or a moderate to large increase that persisted for at least 12 months [10].

In patients with chronic musculoskeletal pain, multidisciplinary or interdisciplinary pain rehabilitation either improved HRQOL, or it did not [10,11,12,13,14]. However, major improvement in HRQOL after pain rehabilitation programs positively associated with shorter duration of pain and worse baseline HRQOL [15]. Similarly, two years after participating in a pain program for patients with mixed chronic musculoskeletal pain, improvements in pain and function were maintained, health-care usage decreased, and the number of working hours increased [16]. Taken together, these findings are suggestive that further study is needed on the effect of pain rehabilitation programs for chronic pain and examination of variables that affect patient outcomes.

One variable known to impact pain is sleep. Sleep problems are frequently reported in adults with chronic pain, and the association is bidirectional [1]. For instance, sleep disorders among patients with low back pain decrease quality of life, and the level of sleepiness is influenced by the intensity of pain [17]. Among individuals with rheumatic diseases, feeling rested after sleep and having a good sleep structure predict better HRQOL outcomes [18]. When pain and sleep are comorbid, both must be addressed to reap the maximum response to pain rehabilitation programs [19].

With its 360,000 inhabitants, Iceland has three main multidisciplinary long-term pain rehabilitation programs. Only a single study has examined the effects of these programs on chronic pain, HRQOL, and sleep. Women with chronic pain who participated in a rehabilitation program that offered either traditional multidisciplinary pain management or neuroscience education and mindfulness-based cognitive therapy were studied. The results indicate that both programs improved quality of life and reduced pain intensity [20] and that the improvements lasted six months after the program’s completion [21]. No Icelandic study focusing specifically on the long-term effects of these programs on chronic pain, sleep, and HRQOL was found.

The aim of the present study was to investigate the long-term effects of multidisciplinary pain rehabilitation interventions in Iceland by (a) exploring and describing how individuals with chronic pain evaluate pain severity, sleep, and HRQOL pre-treatment and at one-year follow-up and by (b) determining those factors that predict the participants’ HRQOL one year after the intervention.

## 2. Materials and Methods

This longitudinal prospective cohort study aimed to investigate pain severity, sleep, and HRQOL in a sample of people with chronic pain undergoing a multidisciplinary pain rehabilitation intervention. Questionnaires were used at two different time points: pre-treatment and at one-year follow-up.

### 2.1. Participants

Participants were men and women in one of three Iceland pain rehabilitation centers. The emphasis of the study was on the intervention that the participants were to receive. Based on recommendations from the nurse unit managers and chief physicians in each center, patients who did not attend the entire program, who participated in a distance program, and who had cancer were excluded from the study. The inclusion criteria for participation were chronic musculoskeletal pain lasting at least three months; the ability to speak, understand, and read Icelandic; an age of 18–70 years (the investigated treatments are not offered to people older than 70 years of age); and admission to one of the three investigated rehabilitation centers.

The reasons for exclusion and withdrawal were not systematically assessed. However, several reasons for exclusion were reported, such as not meeting the inclusion criteria due to a cancer diagnosis, program postponement, removal from the waiting list, not completing the program, and transferring to a distance program or another type of program. Those who withdrew from the study but met the inclusion criteria reported reasons such as not wanting to participate, sickness, not feeling up to it, inability to complete online questionnaires, and uncertainty as to whether they would attend the program.

Permission to conduct the study was granted by the Icelandic National Bioethics Committee (VSN-15-101 on 3 July 2015) and the chief physicians at the three investigated rehabilitation centers. The introductory letter given to the participants included information on the responsible parties and contact persons able to address their questions, comments, or concerns. The methodology was explained, and the respondents were informed about their right to withdraw from the study at any time.

### 2.2. Intervention

The intervention in the present study was a multidisciplinary pain rehabilitation program offered at three rehabilitation centers. These centers are staffed with nurses, physicians, physiotherapists, psychologists, occupational therapists, social workers, nutritional consultants, massage therapists, and physical activity instructors. The standard intervention was similar in all three centers, and treatment lengths ranged from four (centers 2 and 3) to seven weeks (center 1). The intervention began and ended with assessing each patient’s condition. At the initial assessment, every patient was asked to set goals and make decisions regarding the development of further rehabilitation procedures. The standard intervention included scheduled individualized and group sessions comprising physical therapy, cognitive behavioral therapy, relaxation, aquatic exercise training, support, and education. The emphasis of the education piece regarding different subjects related to pain and pain management, such as healthy lifestyle choices, goal setting, relaxation, stress management, sleep, medication, physical training, self-image, and coping. Two of the investigated centers (1 and 3) also offered mindfulness, massage, acupuncture, body awareness, and compassion-focused therapy.

As described above, the three investigated centers offer similar (albeit not identical) multidisciplinary interventions. Due to the emphasis on standard multidisciplinary interventions, the small number of participants, and the variety of causes of chronic pain, it was decided that the participants would be addressed as one cohort.

### 2.3. Procedure

The patients (*n* = 380) were screened according to the inclusion criteria by a contact person at each center (either the chief physician or the nurse unit manager) as soon as they were added to the waiting list for the program. Incoming patients who fulfilled the inclusion criteria (*n* = 236) then received a phone call from a research assistant who introduced the study and provided instructions on how to participate. Those who agreed to participate received an introductory letter by mail with a link and password that enabled them to access and complete a questionnaire online. Those who responded to the first questionnaire (*n* = 144) received a second questionnaire (also online). A reminder was sent by email to those who did not respond within two weeks, a second reminder was sent a week later if there was still no response, and a final reminder was sent four weeks later. During the data-collection process, 31 patients withdrew from further participation, and 32 were excluded. The data were collected between September 2015 and February 2019.

The study questionnaires were based on questionnaires that had been used in another study in Iceland [4] but also included questions developed specifically for this study. The questionnaires measured sociodemographic information, pain severity, pain characteristics, sleep, and HRQOL.

### 2.4. Sociodemographic Information

Demographic information was collected pre-treatment and included age (years), gender (male or female), education (compulsory, upper secondary, or higher), employment status (full-time, part-time, or other), marital status (married or living with a partner, engaged not living together, single, divorced, or widowed), and number of children younger than 18 years of age.

### 2.5. Pain Duration, Causes, and Pain Severity

The participants were asked to report how long they had been in pain (years/months). They were also asked to indicate what they perceived to be the primary cause of their pain and whether they had been diagnosed or had an explanation for the cause of their pain (yes/no). Those who responded “yes” were asked to choose the cause of their pain from a list of possible causes (e.g., accidents, fibromyalgia, disc prolapse, and myalgia).

Pain severity was measured using the Icelandic version of the Brief Pain Inventory (BPI) [22,23]. The BPI includes four questions regarding pain severity (worst, least, average, and pain now). The participants rated their pain severity on a 11-point scale (0 = “no pain,” and 10 = “the worst pain imaginable”).

### 2.6. Sleep

Quality of sleep and sleep problems were measured using four questions developed specifically for this study. The participants were asked to rate their quality of sleep over the past four weeks. The response options were (1) “I had no sleep problems at all,” (2) “I had some sleep problems,” (3) “I had many sleep problems,” and (4) “I had severe sleep problems.” Those who had experienced sleep problems (some, many, or severe) in the previous month were asked to report the reasons. The response options were (1) “because of pain,” (2) “because of other physical problems,” (3) “because of having to get up to use the bathroom,” (4) “because of psychological problems,” (5) “because of noises,” (6) “because I was too cold or too hot,” and (7) “because I sleep in an uncomfortable bed.”

Next, the participants were asked about the effect of their self-perceived sleep problems on their daytime energy. The response options were (1) “No,” (2) “Yes, in some way,” and (3) “Yes, I am extremely tired and have difficulties dealing with daily activities.”

Finally, they were asked how often or rarely they had experienced the following over the past four weeks: (a) “I had trouble falling asleep,” (b) “I used tranquilizers to sleep,” (c) “I used painkillers to sleep,” (d) “I napped during the day,” (e) “I woke up feeling rested,” (f) “I woke up during the night,” (g) “I slept through the night,” and (h) “I used sleep medication.” The response options were (1) “never,” (2) “1–3 times per month,” (3) “1–3 times per week,” (4) “4–6 times per week,” and (5) “daily.” Sleep problems (many and severe sleep problems) and using medication to fall asleep were considered regular if participants reported a frequency of 4–7 times per week.

### 2.7. Health-Related Quality of Life

The Short-Form 36 Health Survey version 2 (SF-36v2) questionnaire comprises multiple-choice questions, and the reliability and validity of the instrument has been tested and confirmed [6,24]. The SF-36v2 is aggregated into eight dimensions: (1) physical function (PF, 10 questions), (2) role physical (RP, 4 questions), (3) bodily pain (BP, 2 questions), (4) general health (GH, 5 questions), (5) vitality (VT, 4 questions), (6) social functioning (SF, 2 questions), (7) role emotional (RE, 3 questions), and (8) mental health (MH, 5 questions) [25,26].

Together, the outcomes of four of the dimensions (PF + RP + BP + GH = 10 + 4 + 2 + 5 = 21 items) constitute the physical component summary (PCS), while the sum of the other four (VT + SF + RE + MH = 4 + 2 + 3 + 5 = 14 items) form the mental component summary (MCS). The responses vary from “Yes, limited a lot”/“Yes, limited a little”/“No, not limited at all” to five-point (“None of the time” to “All the time”) or six-point (“Nothing” to “Very much”) verbal rating scales depending on the original source of the questions [24]. Standardized scores range from 0 to 100 for each dimension [6], with lower scores indicating worse health status (e.g., greater fatigue).

### 2.8. Statistical Analysis

Statistical analysis was conducted using SPSS 27 statistical program (IBM SPSS Statistics for Windows, Version 27.0. Armonk, NY, USA: IBM Corp) [27]. Descriptive statistics (mean, standard deviation, and percentages) were used to present the sample’s demographic information and sleep status at pre-treatment and one-year follow-up. A paired *t*-test with bootstrapping was used to detect differences in pain severity between pre-treatment and one-year follow-up. A related-samples McNemar change test was used to detect the difference in sleep quality between pre-treatment and one-year follow-up. A paired *t*-test bootstrap was used to compare the differences in HRQOL. Differences in pain severity and differences in HRQOL were interpreted using Cohen’s *d* as small (0 to 0.2), medium (0.3 to 0.7), and large (>0.8). The level of significance established for this study was set at *p* < 0.05.

We estimated how well several factors predicted PCS and MCS at one-year follow-up by means of multiple linear regression (separate models were constructed for PCS and MCS). Five variables were introduced into each model to explore their connection to the outcome of the multidisciplinary pain rehabilitation intervention. After searching the literature for variables related to quality of life after multidisciplinary intervention, a decision was made to put these five variables in the regression models. The pre-treatment PCS score was entered into the model for PCS at one-year follow-up, and the pre-treatment MCS score was entered into the model for MCS at one-year follow-up. The sociodemographic variables of being female, having pursued higher education, and having children younger than 18 years of age were entered into both models, along with the variable having many or severe sleep problems at one-year follow-up.

## 3. Results

### 3.1. Characteristics of the Sample (n = 79)

In the end, the study comprised participants who completed both questionnaires (*n* = 79). A nearly equal number of participants attended the intervention in Center 1 (*n* = 39) and Center 2 (*n* = 36), while *n =* 4 participants attended Center 3. The respondents’ ages ranged from 20 to 68 years (*M* = 47.4, *SD* = 11.9 years). Most of the respondents were women (85%), 27% had completed higher education, and 36% were working (23% full time and 13% part time). Most of the participants were married or living with a partner (71%), and 57% had children younger than 18 years of age. The participants’ pre-treatment sociodemographic characteristics are listed in Table 1.

### 3.2. Pain Duration, Causes, and Pain Severity

The mean pain duration was 10.3 years (range 1–55 years). Before treatment, most of the participants (*n* = 72) reported that they had received an explanation or diagnosis for their pain. The most frequently reported perceived causes were fibromyalgia (*n* = 39), accidents (*n* = 35), myalgia (*n* = 33), and disc prolapse (*n* = 24).

The participants rated their pre-treatment pain severity (0–10) higher than at one-year follow-up. The average self-reported pain severity decreased significantly from pre-treatment to one-year follow-up (*p* < 0.001) (medium effect). In addition, there was a significant reduction in the self-reported estimates of the worst pain (*p* = 0.041) and pain now (at the time of the survey) (*p* = 0.048) from pre-treatment to one-year follow-up (small effect). The differences in self-reported pain severity are listed in Table 2.

### 3.3. Sleep Problems

Most of the participants reported disrupted sleep both before the treatment and at one-year follow-up. At pre-treatment, two of the most common reasons for having sleep problems were pain (89%) and psychological problems (49%). At one-year follow-up, the prevalence of these reasons did not change either for pain (*p* = 0.227) or for psychological problems (*p* = 0.541).

As shown in Table 3, the only significant difference in sleep was that more participants (*n* = 5, 6% vs. *n* = 14, 18%) slept through the night at one-year follow-up (*p* = 0.004).

### 3.4. Health-Related Quality of Life

At one-year follow-up, HRQOL had increased. The mean PCS scores were higher than before treatment (*p* < 0.001), and the scores of all PCS subgroups increased significantly, with medium effect except for general health (small effect). The mean MCS score increased but not significantly (*p* = 0.123). The scores of two of the MCS subgroups, VT (*p* = 0.011) and SF (*p* = 0.038), increased significantly, although those of the other two subgroups, RE (*p* = 0.117) and MH (*p* = 0.060), did not increase (Table 4).

### 3.5. Predictors for Differences in MCS and PCS Scores

Two regression models are presented. The first model examined predictors of PCS, and the second model evaluated predictors for MCS at one-year follow-up, as shown in Table 5.

Model 1 explained 23% of the variance (adjusted *R*^2^ = 0.23, *p* < 0.001). The model included PCS at pre-treatment, being female, having pursued higher education, having children younger than 18 years of age, and having many or severe sleep problems at one-year follow-up (sleep problems at one-year follow-up). The only single variable that was a significant predictor for a higher PCS score at one-year follow-up was a higher PCS score at pre-treatment (Table 5).

Regression model 2 was also significant (*p* < 0.001) and explained 46% of the variance (adjusted *R*^2^ = 0.46). The model included MCS at pre-treatment, being female, having pursued higher education, having children younger than 18 years of age, and having sleep problems at one-year follow-up. The pre-treatment MCS was a significant predictor of the one-year follow-up MCS. Being a female, having children younger than 18 years of age, and having sleep problems at one-year follow-up predicted a lower MCS score at one-year follow-up, while having pursued higher education predicted a higher MCS score (Table 5).

## 4. Discussion

The aim of the current study was to investigate the long-term effects of a multidisciplinary pain rehabilitation intervention offered by three main programs in Iceland by exploring and describing how individuals with chronic pain evaluate pain severity, sleep, and HRQOL pre-treatment and at one-year follow-up. The results indicated that the intervention for the participants decreased pain severity and increased HRQOL. Comparison of pre-and post-treatment scores revealed some small effect size with significant *p* values in pain reduction. This finding is in agreement with the systematic review of Salathé et al. on studies that examined pain intensity over 12 months (either with VAS or NRS) following multidisciplinary biopsychosocial rehabilitation. Comparison of pre-and posttreatment scores revealed either moderate to large effect size with significant *p* values in pain reduction. In assessing patients over a longer period, they showed that the reduction in pain intensity persisted for at least 24 months [10]. In the current study, although the pain severity scores decreased significantly, pain was still high (around 6), and pain did disturb sleep and HRQOL. A reduction of average pain was only 0.7 points over one-years’ time, which would not be concluded as a clinically important difference for pain if compared to the results of Mease et al.’s study [28], which shows that the anchor-based minimum clinically important difference (MCID) for the BPI average pain and severity scores for fibromyalgia were 2.1 and 2.2. points, which correspond to 32.3% and 34.2% reduction from baseline in scores.

Multiple pain causes combined with a long duration of pain have been associated with poor quality of life [29]. In the current study, the participants’ mean length of years in pain was 10.3 years (ranging from 1 to 55 years), and some of the participants had more than one perceived cause of pain. At one-year follow-up, HRQOL increased, especially the PCS. In Vartiainen et al.’s study, 81% experienced a major improvement, and 12% felt no change in HRQOL after a pain rehabilitation program [15]. A shorter duration of pain (<3 years) was positively associated with major improvement. In that same study, pain intensity was measured with VAS (0–100). There was no significant change in pain intensity at 12-month follow-up. HRQOL was measured with 15-D score, and the mean score of the patient in the total sample increased by 0.017 (from 0.711 to 0.728), which was a clinically important mean change.

In the current study, the mean score in all PCS subgroups significantly increased, which has been observed in other studies as well [11,12]. However, the mean scores increased significantly only in the VT and SF subgroups of MCS at one-year follow-up were during the previous month and whether their physical and emotional health interfered with communication with family and friends. Having less energy and being tired can be associated with having sleep problems; 32% of the participants in this study responded that their sleep problems affected their daytime energy, and 48% had many or severe sleep problems pre-treatment. In the second aim of the current study, we wanted to determine the factors that predicted the participants’ HRQOL one year after the intervention.

Having sleep problems was one of the predictive variables of the MCS. Other findings indicate that chronic pain makes people more likely to suffer from sleep problems, depression, and other psychiatric disorders [30,31,32].

Sleep deprivation has been found to be a risk factor for chronic pain in a 17-year survey of women [33]. In the current study, self-reported reasons for sleep problems showed some interesting results. Concerning sleeping through the night, significant differences in sleep quality were found between pre-treatment and one-year follow-up, although only 18% of the respondents slept through the night at one-year follow-up. Although pain severity decreased and HRQOL increased, pain and psychological troubles were still the main reasons for sleep problems. This is in accordance with other studies [30]. Even though the focus of the intervention has been on sleep, this raises the question of whether enough was done in the intervention to deal with sleep problems in connection with pain and psychological troubles.

In the current study, higher education predicted higher MCS scores, a finding that is supported by other studies [14,15,17,18]. The reasons for this finding are not clear, but it may be that higher education makes people more open to new ideas, or those with higher education are more likely or more able financially to engage in better self-care after completing pain rehabilitation programs.

One unexpected result was that being female predicted lower MCS scores in our study. Previous studies exploring multidisciplinary pain-treatment programs demonstrate that women improved more than men [11,14]. It is known that women usually participate in similar studies more than men [11,14,16], and women are more likely to report or experience pain and to seek treatment for their pain [1,4]. Having children younger than 18 years of age also predicted lower MCS score. No previous studies were found with similar results. Perhaps the responsibility of having young children at home affects women’s mental health, energy, and sleep. Further studies are needed to explore these differences.

Although mental health issues were not the main focus of the current study, we found that participants who reported feeling anxious and depressed showed very little improvement following the intervention. It is well known that patients with severe pain are more likely to be depressed [30,34] and that depression is often unrecognized and untreated [31]. There is a bidirectional relationship between chronic pain and mental health conditions [1], and depression, anxiety, and negative beliefs about pain are all related to developing pain and having worse outcomes from chronic pain [1]. Patients with chronic pain should be examined with respect to their mental status [30], and more follow-up is needed after the completion of a pain rehabilitation intervention to deal with mental health problems.

## 5. Strengths and Limitations

A major strength of the current study is its examination of three similar pain rehabilitation programs in the country of Iceland. Albeit not identical, the standard programs shared similarities in the emphasis. An important finding was that the interventions were effective for the participants. It is possible that our findings apply to similar interventions in countries with larger populations and similar ethnic backgrounds, but further studies are needed.

We did not use the smallest difference scores in the domain of interest that chronic pain patients perceive beneficial (Minimal Clinical Important Difference (MCID) or Minimum Detectable Change (MDC)). The effect size of difference in pain severity and HRQOL was small to medium. Sleep quality did not change at one-year follow-up, and most participants had disrupted sleep because of pain. Their use of tranquilizers, pain killers, or sleep medication had not changed significantly. Use of medication for pain and sleep is another area that requires further study.

We did not use experimental design with control groups because it was not feasible to deny some of the participants treatment. This makes it impossible to make statements about direct cause and effect, which is a limitation of the study.

A main limitation of this study was the small number of participants and the composition of the subjects. Fewer men than women participated, which made it difficult to perform gender comparisons. The length of the standard program varied from four to seven weeks, which is a limitation. Furthermore, in two of the centers, there were some health disciplines not offered in the third program. When searching for an effect of an intervention of patients’ pain, sleep, and health-related quality of life, it is easier to conclude about effect of the intervention if everyone participated in the same intervention in the same period for the same amount of time with exactly the same health disciplines. We did not have access to waiting lists or list of incoming patients, so we could not anticipate potential subject recruitment. Additionally, this group of participants was complex: multiple causes of pain, length of pain, and varied backgrounds of the subjects may have contributed to a smaller treatment effect. Another limitation was that the intervention was scheduled for each individual, so the whole group did not necessarily attend the same number of hours in the standard program. It is possible that the intervention would have been more beneficial if subjects were treated earlier in their pain experience and with equivalent hours for the intervention. We did not evaluate the level of support each subject had, and support may be an important variable for future study. It is logical to assume that increased support would contribute to the long-term success of the intervention.

## 6. Conclusions

The results indicated that multidisciplinary pain rehabilitation program of three major centers in Iceland was effective in decreasing pain severity and increasing HRQOL one year after completing the intervention with a small to medium effect. PCS scores increased significantly, and the pre-treatment PCS score predicted the one-year follow-up PCS score. Patients should be examined with respect to their mental status and sleep problems, and it would be beneficial if they received professional support after completing the intervention. Pre-treatment MCS scores and having pursued higher education predicted higher MCS scores at one-year follow-up. However, having many or severe sleep problems, being a woman, and having children under 18 years of age predicted lower MCS scores at one-year follow-up. Sleep was still disturbed by pain and psychological problems at one-year follow-up, although more participants slept through the night than before treatment. These findings support the effectiveness of multidisciplinary rehabilitation programs for pain and will be used to guide further research in pain therapeutics.

## Figures and Tables

**Table 1:** Description of sociodemographic variables (*n* = 79).

Variables	*n*	%
**Gender**		
Females	67	85
Males	12	15
**Age**		
43 years or less	27	34
44–54 years	26	33
55 years or older	26	33
**Education**		
Compulsory	26	33
Upper secondary	30	38
Higher	21	27
**Marital status**		
Married/living with a partner	56	71
Engaged not living together	10	13
Single/divorced/widowed	12	15
**Employment status**		
Full time	18	23
Part time	10	13
Other	54	64
**Children < 18 years**	43	57

**Table 2:** Pain severity (*n* = 79).

	Pre-Treatment *M* (*SD*)	One-Year Follow-Up *M* (*SD*)	*p*-Value *	Cohen *d*
Pain severity				
Worst now	7.4 (1.78)	6.8 (2.08)	**0.048**	0.23
Worst	8.4 (1.56)	7.9 (1.97)	**0.041**	0.23
Least	4.5 (1.93)	4.4 (2.01)	0.517	0.07
Average	6.6 (1.65)	5.9 (1.83)	**<0.001**	0.42

*M*, mean; *SD*, standard deviation. * Values in bold indicate statistically significant differences.

**Table 3:** Sleep quality (*n* = 79).

Sleep Quality	Pre-Treatment *n* (%)	One-Year Follow-Up *n* (%)	*p*-Value *
I had many or severe sleep problems	38 (48.1)	28 (35.4)	0.078
How often has the following happened over the last month?			
A. I had trouble falling asleep	30 (38.9)	25 (32.4)	0.383
B. I used tranquilizers to sleep	19 (26.4)	21 (29.2)	0.791
C. I used pain killers to sleep	23 (30.6)	22 (29.3)	1.00
D. I napped during the day	11 (14.6)	8 (10.6)	0.581
E. I woke up feeling rested	4 (5.3)	5 (6.6)	1.00
F. I woke up during the night	49 (63.6)	48 (62.3)	1.00
G. I slept through the night	5 (6.7)	14 (18.7)	**0.004**
H. I used sleep medication	11 (14.5)	8 (10.5)	0.453
Effect of sleep problems on daytime energy	23 (32.3)	16 (22.5)	0.143

* Values in bold indicate statistically significant differences.

**Table 4:** The mean difference in HRQOL pre-treatment and at one-year follow-up (*n* = 79).

	*n*	Pre-Treatment *M* (*SD*)	One-Year Follow-Up *M* (*SD*)	Pre/One-Year *p*-Value *	Cohen *d*
PCS (physical component summary)	78	33.0 (6.2)	36.2 (6.9)	**<0.001**	0.48
PF (physical functioning)	79	37.5 (7.8)	40.6 (7.8)	**0.002**	0.35
RP (role physical)	79	28.1 (6.4)	32.3 (6.9)	**<0.001**	0.46
BP (bodily pain)	78	30.2 (5.7)	34.3 (7.6)	**<0.001**	0.55
GH (general health)	79	38.5 (8.4)	40.5 (9.2)	**0.039**	0.24
MCS (mental component summary)	78	38.3 (10.8)	40.1 (11.3)	0.123	0.18
VT (vitality)	78	34.4 (6.3)	36.9(8.0)	**0.011**	0.31
SF (social functioning)	78	34.3 (9.05)	36.6 (9.7)	**0.038**	0.24
RE (role emotional)	77	34.9 (12.8)	37.5 (11.4)	0.117	0.17
MH (mental health)	77	39.9 (9.9)	42.1 (10.0)	0.060	0.22

*M*, mean; *SD*, standard deviation. * Values in bold indicate statistically significant differences.

**Table 5:** Regression models 1 and 2 of potential predictors of PCS and MCS at one-year follow-up.

	B	95% CI for B		*t*	*p*-Value *	Adjusted *R*^2^	*F*
Model 1 PCS at one-year follow-up							
(Constant)	18.21	11.01	25.16	4.24	**0.001**	0.23	5.53
Pre-treatment PCS	0.49	0.29	0.72	4.29	**0.001**		
Female	2.33	−0.98	5.35	1.19	0.131		
Children < 18 years	0.27	−2.45	2.97	0.19	0.838		
Higher education	0.39	−3.17	4.03	0.25	0.832		
Sleep problems at one-year follow-up	−2.08	−4.77	0.39	−1.40	0.111		
Model 2 MCS at one-year follow-up							
(Constant)	28.60	19.23	38.71	6.18	**0.001**	0.46	14.23
Pre-treatment MCS	0.49	0.29	0.70	5.44	**0.001**		
Female	−5.20	−9.59	−0.95	−1.96	**0.016**		
Children < 18 years	−4.04	−7.57	−0.36	−2.08	**0.040**		
Higher education	4.71	0.21	9.29	2.16	**0.045**		
Sleep problems at one-year follow-up	−6.20	−10.49	−2.18	−3.02	**0.006**		

* Values in bold indicate statistically significant differences.
